# A Comparative Study on Tendon Transfer Surgery in Patients with Radial Nerve Palsy 

**Published:** 2014-01

**Authors:** Masoud Yavari, Hossein Ali Abdolrazaghi, Azadeh Riahi

**Affiliations:** 1 Department of Hand Surgery, Shahid Beheshti University of Medical Sciences, Tehran, Iran;; 2 Department of Occupational Therapy, 15th Khordad Hospital, Tehran, Iran

**Keywords:** Tendon transfer, Radial nerve palsy, Flexor carpi ulnaris, Flexor carpi radialis, Palmaris longus, Pronator teres

## Abstract

**BACKGROUND:**

Lesions in peripheral nerves are highly prevalent in the upper extremity. The present study compares different tendon transfer surgeries in patients with radial nerve palsy.

**METHODS:**

Fifty patients with radial nerve palsy were randomly selected among patients who referred to Tehran 15^th^ Khordad Hospital during 2006-2011. They were divided into two groups of 17 and 33 subjects. Single tendon transfer surgery was performed on 33 and ternary tendon transfer surgery on 17 patients and were compared.

**RESULTS:**

No significant difference was noticed in the range of motion of metacarpophalangeal joint, proximal interphalangeal joint and distal interphalangeal joint joints between the two groups. There was also no significant difference in the results of single tendon and ternary tendon transfer surgeries between the two groups. There was no need to sacrifice three tendons in tendon transfer surgeries on patients with radial nerve palsy.

**CONCLUSION:**

Single tendon transfer surgery may help establishing a finger extension while indicates to its considerable advantages of surgical simplicity, shorter surgery time, less complications and surgery scars.

## INTRODUCTION

Upper extremity lesions are highly prevalent so that almost 1.3% of body lesions occur in these limbs. Lesions in peripheral nerves including median, ulnar and radial nerves are highly prevalent in the upper extremity. So many patients with hand lesions refer to hospitals and rehabilitation centers while among the peripheral nerves, radial nerve is more vulnerable to damage.^1^

The treatment method for radial nerve palsy depends on the primary cause and the severity of damage. Radial nerve palsy without associated laceration or perforation is considered as closed nerve palsy. Humerus fractures may result in closed radial nerve lesions that are usually observed for a 3-month period before any surgery. Unknown symptoms of radial nerve palsy may undergo cautious treatment following the rejection of curable causes such as tumors. Surgical methods for the treatment of open radial nerve lesions, which damage the nerve pathway, are nerve repair and nerve or tendon transfer.^1^

In tendon transfer surgery, which is done to restore the missing function and performance, a tendon is transferred to the junction of a muscle-tendon unit. Most researchers contend that tendon transfer in patients with radial nerve palsy may bring about good results once nerve repair has failed. When no improvement in radial nerve lesions is noticed within a one-year interval, tendon transfer may be recommended.^2^

In the early 1900s, the use of flexor carpi ulnaris was developed in order to restore the extension of metacarpophalangeal joint, though there are obstacles to the use of flexor carpi ulnaris.^3^^,^^4^ For example, the use of flexor carpi ulnaris would sacrifice the only operating agent that moves the wrist towards ulnar resulting into the radial deviation of the wrist, particularly in the case of moderate radial nerve palsy in which extensor carpi radialislongus remains intact. Besides, when transferring flexor carpi ulnaris, ulnar deviation with the wrist flexion is lost. This is an important wrist movement, which is necessary for activities such as hammering and throwing. Therefore, instead of flexor carpi ulnaris, flexor carpi radialis and flexor digitorum superficialis are often transferred.^5^

Once transferred, neither flexor carpi radialis nor flexor digitorum superficialis do cause the wrist radial deviation or the loss of ulnar deviation with wrist flexion. The transfer of flexor digitorum superficialis is advantageous over the other two alternatives because it provides better sliding. This is particularly important in patients with wrist fusion who cannot use tenodesis effect. The major problem with the transfer of flexor digitorum superficialis is that the power extension of metacarpophalangeal joint is not synergic with finger flexor, which makes the motor retraining difficult following the transfer.^6^

Over the last century, three methods were developed for tendon transfer in radial nerve palsy including the transfer of flexor carpi radialis (Brand),^4^ the transfer of flexor carpi ulnaris and the transfer of superficialis (Boyes).^6^ In these three methods, pronator teres is transferred to extensor carpi radialis brevis in order to restore the wrist extension; however, these methods differ when used to restore the extension in thumb and fingers. The transfer of flexor carpi radialis includes the transfer of flexor carpi radialis to extensor digitorum communis in order to restore finger extension as well as the transfer of Palmaris longus to extensor pollicis longus in order to restore thumb extension.^7^^,^^8^

The transfer of flexor carpi ulnaris follows the same procedure except for the fact that flexor carpi ulnaris is transferred to flexor carpi radialis to reinforce extensor digitorum communis. The transfer of superficialis is somewhat different from other cases. Flexor digitorum superficialis of the ring finger is transferred to extensor pollicis longus and extensor indicis proprius in order to restore the extension of thumb and index finger. Flexor digitorum superficialis of the middle finger is used to both restore the extension of fingers and to reinforce extensor digitorum communis of all fingers. Besides, flexor carpi radialis may be transferred to abductor pollicis longus and extensor pollicis brevis in order to restore the independent radial abduction of the thumb. Each of these transfers has proved effective in various distributions. There is no comparison to account for the relative advantage of one method over the other.^9^^-^^12^

The fourth and last method of tendon transfer is Merle d’Aubigne procedure. As with other methods, it includes the transfer of pronator teres to extensor carpi radialis brevis (and extensor carpi radialis longus). As well, flexor carpi ulnaris is used to reinforce both extensor pollicis longus and extensor digitorum communis. Moreover, Palmaris longus is transferred to extensor pollicis brevis and abductor pollicis longus in order to restore thumb abduction. This alternative has also been approved as a valid meth od in treating radial nerve palsy.^13^

Although surgeons frequently use different methods in tendon transfer, no research has yet addressed the potential advantage of one method over the other. Therefore, search for an appropriate method of tendon transfer may help alleviate sensory-motor problems in the patients following the treatment and reduce the costs imposed upon the industries, particularly considering the high prevalence of nerve lesions such as radial nerve damages. In this regard, we set to compare the efficiency of two methods of tendon transfer including ternary tendon transfer (palmaris longus/flexor carpi ulnaris/flexor carpi radialis) and single tendon transfer (flexor carpi ulnaris) in improving the performance of upper extremity in patients with radial nerve palsy.^14^^-^^16^

## MATERIALS AND METHODS

Fifty patients with radial nerve palsy were randomly selected among the patients who referred to Tehran 15^th ^Khordad Hospital during the years 2006-2011 and assigned into two groups of 17 and 33 patients. A single tendon transfer surgery was performed on the 33 patients group and ternary tendon transfer surgery on the 17 subjects group. The study was approved in Ethics Committee of our institution and all patients completed an informed consent form. Following the completion of a demographic data sheet by the patients, the single tendon transfer and ternary tendon transfer surgeries were undertaken. SPSS software (Version 11, Chicago, IL, USA) was used for statistical analysis. With regard to the independent variables and research hypotheses, inferential statistics including independent t test, Mann Whitney U test and ANOVA were used to analyze the data. The range of motion was considered for metacarpophalangeal joint (MP), proximal interphalangeal joint (PIP) and distal interphalangeal joint (DIP) joints as the average range of motion pertaining to each joint in the fingers. A P value less than 0.05 was considered significant.

## RESULTS

Ranges of motion in metacarpophalangeal joint (MP), proximal interphalangeal joint (PIP) and distal interphalangeal joint (DIP) after single tendon transfer surgery (n=33) and ternary tendon transfer surgery (n=17) were shown in Table 1. There was no significant difference in the range of motion in MP, PIP and DIP joints between the two groups. No significant difference was seen between the results of single tendon transfer and ternary tendon transfer surgeries used to treat radial nerve palsy. No significant difference was visible between the mean scores of both methods and in the ranges of joint motion between the two surgery methods. 

**Table 1:** Ranges of motion in metacarpophalangeal joint (MP), proximal interphalangeal joint (PIP) and distal interphalangeal joint (DIP) after single tendon transfer surgery (n=33) and ternary tendon transfer surgery (n=17).

	**Single tendon transfer surgery**	**Ternary tendon transfer surgery**	**P value**
MP	88.61±63.10	77.29±12.11	0.326
PIP	90.47±40.09	85.94±13.46	0.559
DIP	265.13±15.65	267.32±7.96	0.514

## DISCUSSION

In tendon transfer surgery which is done to restore the missing function and performance, a tendon is transferred to the junction of a muscle-tendon unit. Most researchers reported that tendon transfer in patients with radial nerve palsy may bring about good results once nerve repair has failed. When no improvement in radial nerve lesions is noticed within a one-year interval, tendon transfer may be recommended.^2^

Currently, there is no consensus on the best method for tendon transfer in patients with radial nerve palsy. The severity of radial nerve damage as well as the anatomy and general performance of a patient often determines the best available surgery method. However, surgeons use different combinations of tendon transfers in order to achieve three major goals in the treatment of radial nerve palsy including restoration of finger extension (metacarpophalangeal joint), restoration of thumb and wrist extension in cases of sever radial nerve palsy.^2^^,^^7^^,^^17^

Following severe radial nerve damage, the most acceptable method to restore wrist extension is to transfer pronator teres to extensor carpi radialis brevis. When there is no chance for the radial nerve recovery, the transfer should be made via end-to-end method whereby extensor carpi radialis brevis tendon is cut and stitched up to the severed end of pronator teres tendon. This method results in a straight direction of tension and more efficient transfer.^2^

When radial nerve is already repaired and the recovery of extensor carpi radialis brevis is expected to occur in future, the transfer should be made using end-to-side method whereby pronator teres tendon is cut and stitched up to the side of the intact extensor carpi radialis brevis tendon. If this treatment is done early, it will function as an internal splint to fix the wrist while the nerve is recovering. Therefore, since extensor carpi radialis brevis is maintained in good condition, it will restore wrist extension once it has regained its function.^2^^,^^18^

In order to restore thumb extension, several different tendons may be transferred to extensor pollicis longus. To this end, palmaris longus or the flexor digitorum superficialis of the ring finger are often transferred to the extensor pollicis longus. When the flexor digitorum superficialis tendon of the ring finger is used, it can be divided in two and stitched up to both extensor pollicis longus and extensor indicis proprius, which allows the simultaneous extension of both thumb and index finger. Although this transfer seems to violate the principle of using one tendon for each movement, this is not the case.^19^

Simultaneous extension of thumb and index finger is a combined movement that facilitates accurate manipulation and can be considered as a single performance. When palmaris longus is used to generate motion, extensor pollicis longus will usually be redirected towards the volar side to be aligned with palmaris longus in a straight tensile force direction. This causes the abduction of thumb towards the radial as well as the extension of the joints between the fingers (interphalangeal joint). The extension of metacarpophalangeal joints of the fingers can be restored through transferring flexor carpi radialis, flexor carpi ulnaris or flexordigitorum superficialis tendons to the extensor digitorum communis.^2^

In our study, in order to restore finger extension in the patients, 33 subjects underwent single tendon transfer surgery (including the transfer of flexor carpi ulnaris to common extensor digitorum and extensor pollicislongus).The 17 patients in the other group underwent ternary tendon transfer surgery (including the transfer of pronator teres to extensor carpi radialisbrevis, the transfer of flexor carpi ulnaris to common extensor digitorum and the transfer of Palmaris longus to extensor pollicislongus). 

Following one-month postsurgical splinting and physiotherapy, the ranges of motion in MP, PIP and DIP joints were compared between the two groups. The results of statistical analysis showed no significant difference in the ranges of motion in respective joints between the two groups. Accordingly, there was no significant difference between the results of single tendon and ternary tendon transfer surgery. Besides, the results showed that though the primary objective of these surgeries was to restore finger extension, a considerable amount of wrist extension was also regained.^20^^,^^21^

Some researchers reveealed that, in cases of radial nerve injury, the nerve should be first repaired. Then one year interval should be allowed before the patient could be considered as a tendon transfer candidate when the desired result is not attained. However, a new method has been developed whereby if (i) there is more than 4 cm distance between the pieces, (ii) radial nerve palsy is surrounded by deep wound, (iii) radial nerve palsy is surrounded by extensive scar and (iv) radial nerve palsy contains skill loss, then tendon transfer should be immediately done and there is no need for primary radial nerve repair. Consistent with this new method, we conducted immediate tendon transfer surgery on the patients.^7^^,^^11^^,^^17^

Following the removal of splints and the beginning of physiotherapy, on average, the patients could return to work two months after tendon transfer surgery. The point is that both single tendon and ternary tendon transfer surgeries yielded the same results. The findings of the present experiment showed that single tendon transfer could restore finger and wrist extension as well as thumb extension and abduction so that there is no need to transfer more tendons in such surgeries. As a result of the present treatment, patients regained their thumb abduction as much as 60 degrees on average. All patients were able to extend their fingers in the positions of wrist flexion, neutral wrist and wrist extension. They also regained their ability of wrist bending and gripping.^21^

The present findings suggest that there is no need to sacrifice three tendons in tendon transfer surgeries on patients with radial nerve palsy, rather a single tendon transfer surgery may help establish finger extension and press home the considerable advantages of surgical simplicity, shorter surgery time, less complications and surgery scars. It is notable to mention that further studies in near future should be done to compare the grip strength in these patients. 

## Footenote

Please cite this paper as:

Yavari M, Abdolrazaghi HA, Riahi A. A Comparative Study on Tendon Transfer Surgery in Patients with Radial Nerve Palsy. World J Plast Surg 2014;3(1):47-51.
